# Cutaneous Plasmacytosis with Perineural Involvement

**DOI:** 10.1155/2014/840845

**Published:** 2014-02-06

**Authors:** Elizabeth A. Brezinski, Maxwell A. Fung, Nasim Fazel

**Affiliations:** Department of Dermatology, Davis Health System, University of California, 3301 C Street, Suite 1400, Sacramento, CA 95816, USA

## Abstract

*Importance*. Cutaneous and systemic plasmacytosis are rare conditions of unknown etiology with characteristic red-brown skin lesions and a mature polyclonal plasma cell infiltrate within the dermis. Perineural plasma cell infiltrates may be a histologic clue to the diagnosis of cutaneous plasmacytosis. *Observations*. Our patient had a five-year history of persistent reddish-brown plaques on the neck and trunk without systemic symptoms. Histologic examination showed dermal perivascular and perineural plasma cells with excess lambda light chain expression. Due to decreased quality of life caused by his skin lesions, he was placed on a chemotherapeutic regimen with bortezomib. *Conclusions and Relevance*. The patient was diagnosed with cutaneous plasmacytosis based on classic histopathology results with a recently characterized pattern of perineural involvement. Bortezomib therapy was initiated to manage his skin eruption, which has not been previously described as a treatment for this chronic condition.

## 1. Introduction

Cutaneous and systemic plasmacytosis are rare, lymphoplasmacytic disorders characterized by red-brown poorly circumscribed plaques and nodules occurring mainly on the trunk primarily in patients of Japanese descent [1, 2]. The disease can be accompanied by fever, lymphadenopathy, anemia, and a polyclonal hypergammaglobulinemia [2, 3]. The characteristic histopathology is dermal perivascular infiltrates of mature polyclonal plasma cells [2, 4]. Herein we present a Hispanic patient with chronic red-brown macules and plaques on the trunk where these distinctive biopsy findings supported the diagnosis of cutaneous plasmacytosis. We discuss the classical histopathologic features of cutaneous plasmacytosis and evidence for cutaneous-only involvement of this condition.

## 2. Case

**Figure 1:**
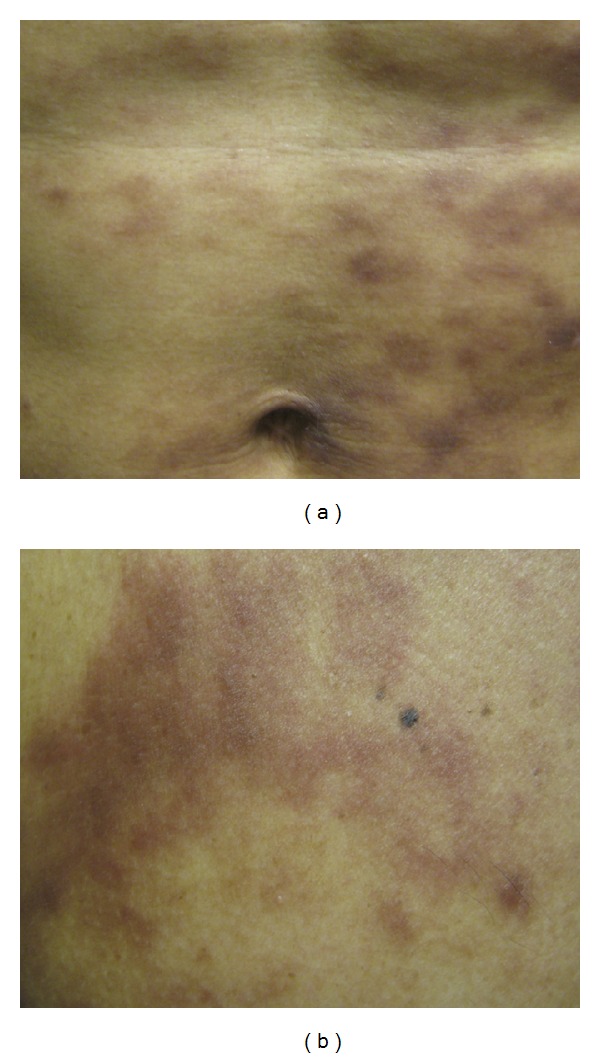
(a) The patient presented with brownish-red macules and mildly indurated plaques on his abdomen. (b) Similar lesions were present on his chest.

A 39-year-old Hispanic man presented with a five-year history of persistent red plaques on his neck and a two-year history of similar lesions that had spread to his trunk, sparing the upper and lower extremities. His past medical history included untreated latent tuberculosis infection and allergic rhinitis. Clinical examination revealed brownish-red macules and mildly indurated plaques on his trunk (Figure 1) and pink-to-violaceous plaques with fine scale on his neck. He was afebrile and had no lymphadenopathy. Laboratory examination showed normal complete blood count, serum protein, and erythrocyte sedimentation rate, no monoclonal protein on immunofixation, and hyperimmunoglobulin (Ig) E (199 KU/L—normal: <25 KU/L). The serum level of interleukin-(IL-) 6 was normal and antinuclear antibodies were negative. Free kappa and lambda chains and the kappa: lambda ratio were normal. Urinalysis was without blood or protein with no measurable Bence Jones protein in the urine. Human immunodeficiency virus, rapid plasma reagin, and Borrelia burgdorferi IgM were negative. Positron emission tomography/computed tomography (PET/CT) showed no evidence of fluorodeoxyglucose (FDG) avid cutaneous lesions or other areas of active disease.

## 3. Histology, Molecular Studies, and Therapeutic Trials

**Figure 2:**
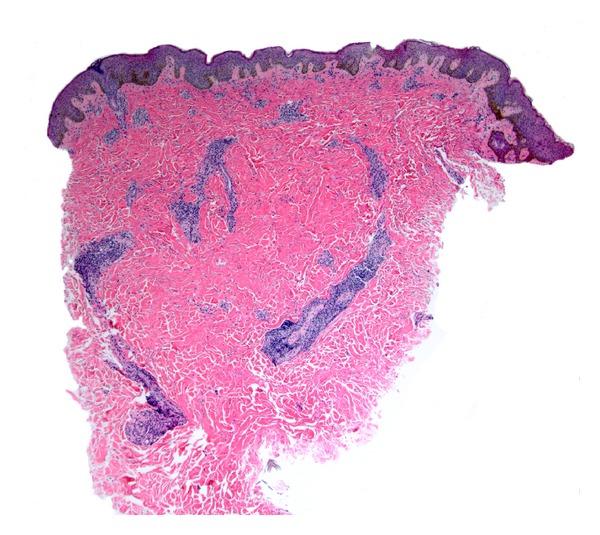
Abdominal biopsy highlighting dermal plasmacytes, which were evident in biopsies of the patient's abdomen, flank, and neck (hematoxylin & eosin stain; original magnification: ×40).

**Figure 3:**
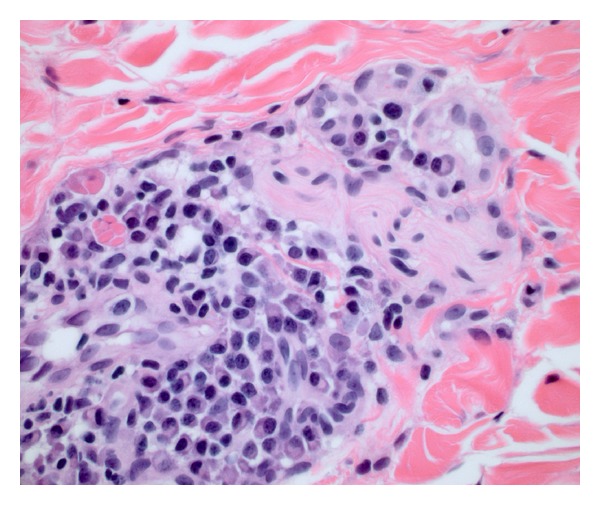
Abdominal biopsy was notable for perineural plasmacytes (hematoxylin & eosin stain; original magnification: ×600).

Skin biopsies from the abdomen, neck/shoulder, flank, and cervical region were obtained. The distinctive feature was a superficial and deep perivascular and perineural dermatitis with prominent plasma cells (Figures 2 and 3). The plasma cells were highlighted by IgG but negative for IgG4. There were evidence of B-cell clonality by PCR gene rearrangement analysis (two of two specimens) and evidence of slight lambda excess by immunohistochemistry and *in situ* hybridization. Other small B cells were highlighted by CD20 but negative for Bcl-6. Small CD3-positive T cells were intermixed with the plasma cells. CD117 marked background levels of mast cells. Immunohistochemical staining for *T. pallidum* was negative. PAS, Fite, and Warthin-Starry stains were negative. Tissue cultures for bacteria, mycobacteria, viruses, and fungi were negative. These skin biopsies were reviewed by our institution's dermatopathologists and hematopathologists and subsequently in consultation at the National Institutes of Health. Bone marrow aspirate demonstrated normocellular marrow with mildly increased numbers of plasma cells seen by CD138 and a minute kappa dominant plasma cell population detected by flow cytometry. Occasional small groups of plasma cells were seen which were predominantly associated with vessels. This distribution with only 5-6% plasma cells in the absence of atypia favored a reactive etiology. Chromosome analysis showed a normal male chromosome complement.

After evaluation by hematology and oncology, the patient was prescribed a one-month course of doxycycline to eliminate a possible nidus of infection with subsequent worsening of his cutaneous plaques on this therapy. He was later treated with a three-week course of clobetasol ointment with continued worsening. Since this patient did not tolerate a trial of clobetasol ointment and there is not currently an FDA-approved pharmacologic therapy for cutaneous plasmacytosis, an alternative therapy was investigated. After extrapolating from cutaneous T- and B-cell malignancy data as well as other plasma cell disorder therapies, the decision was made to start bortezomib. He subsequently was put on subcutaneous bortezomib 1.3 mg/m^2^. His chemotherapy regimen included twice weekly injections over two weeks for a total of four treatments per cycle, which he tolerated with mild nausea and myalgias. The skin lesions did not progress and he did not develop any new eruptions after two cycles of therapy; however, his rash showed partial regression after a total of eight treatments.

## 4. Discussion

Cutaneous plasmacytosis is a disorder of unknown etiology, which was first described by Yashiro as a “kind of plasmacytosis” and later redefined by Kitamura et al. as “cutaneous plasmacytosis” [1, 5]. Whether pure cutaneous plasmacytosis is a condition distinct from systemic involvement has been debated. It has been proposed that asymptomatic patients with manifestations of cutaneous plasmacytosis may have systemic involvement [6, 7]. Thus, these patients may warrant additional work-up, such as a superficial lymph node biopsy, to exclude the disorder of cutaneous and systemic plasmacytosis. Similar to prior case reports of cutaneous plasmacytosis, our patient had no palpable lymphadenopathy to suggest systemic involvement and PET/CT with FDG did not highlight any extracutaneous activity. Further evaluation by bone marrow biopsy revealed slightly increased plasma cells, which was thought to be reactive. It was determined that lymph node biopsy for histopathologic examination to rule out this largely benign, chronic, and indolent condition was not clinically warranted in this patient given the imaging and bone marrow biopsy results and selection of aggressive treatment.

Histopathology characteristically demonstrates a moderately dense, superficial, and deep perivascular and periadnexal infiltrate of plasma cells without atypia [3, 4]. The plasma cells are typically polyclonal and mature. In a minority of cases, accompanying small reactive germinal centers are found [2, 8]. Recently, focal infiltrates of perineural plasma cells were described in six patients with cutaneous plasmacytosis [8]. Intraneural plasma cells were also observed in a subset of these patients. In biopsies from our patient, marked perivascular and perineural plasmacytosis in the dermis was present. After no infectious etiologies were identified, this patient with normal IL-6 and gammaglobulin levels was diagnosed with cutaneous plasmacytosis by clinical correlation between the skin lesions and confirmatory histopathology. This case adds to the spectrum of histopathologic presentations that cutaneous plasmacytosis may take on in the absence of systemic signs and characteristic laboratory findings.

Although this condition is primarily described in middle-aged patients of Japanese descent, there have been case reports of cutaneous plasmacytosis occurring in patients of other Asian ethnicities and Caucasian descent [7]. Our patient is the first Hispanic individual reported to have cutaneous plasmacytosis.

Cutaneous plasmacytosis typically follows a benign clinical course, which does not require treatment; however, decreased quality of life due to the appearance of the lesions was a significant concern for this patient. Various treatment approaches have been tried to induce clinical remission. Topical and systemic corticosteroids, topical tacrolimus, antibiotics, and systemic chemotherapy have produced variable long-term clinical results [3, 9]. Treatment with intralesional steroids, combination prednisone and cyclophosphamide, photodynamic therapy with long-pulse ruby laser, psoralen-UVA, and topical pimecrolimus have reportedly improved skin lesions of cutaneous plasmacytosis [10–14].

Bortezomib is FDA-approved for the treatment of multiple myeloma and mantle cell lymphoma in patients who have received at least one prior therapy [15]. This chemotherapeutic agent acts by inhibiting the 26S proteasome, which prevents selective proteolysis and can lead to cell death. The pathophysiology of cutaneous plasmacytosis has not yet been elucidated and it is uncertain whether therapy that targets other plasma cell malignancies will be effective for plasmacytosis. This patient was placed on a regimen of twice weekly subcutaneous bortezomib followed by a 10-day rest period. After two cycles, the patient developed no new lesions and the persistent plaques had evidence of regression. Additional cycles of chemotherapy are planned and longer-term follow-up is needed. The patient experienced a mild adverse reaction during the course of therapy.

## 5. Conclusion

Cutaneous and systemic plasmacytosis are largely benign conditions with unknown pathophysiology that occur mainly in Japanese patients and can have a wide range of clinical and histopathologic presentations. We report a case of cutaneous plasmacytosis with distinctive perineural involvement, a histopathologic feature that may aid in the diagnosis of this disorder when used with clinical correlation. This is also the first case of a Hispanic patient presenting with cutaneous plasmacytosis. Further, this patient was treated with bortezomib with partial improvement after two cycles of therapy. Additional long-term studies are needed to determine the potential efficacy of targeted chemotherapeutic medications for the management of cutaneous plasmacytosis.

## Abbreviations

Ig: Immunoglobulin
IL: Interleukin
PET/CT: Positron emission tomography/computed tomography
FDG: Fluorodeoxyglucose.
