# Corrigendum to “Cutaneous Plasmacytosis with Perineural Involvement”

**DOI:** 10.1155/2016/8614078

**Published:** 2016-07-04

**Authors:** Elizabeth A. Brezinski, Maxwell A. Fung, Nasim Fazel

**Affiliations:** Department of Dermatology, Davis Health System, University of California, 3301 C Street, Suite 1400, Sacramento, CA 95816, USA

 In the article titled “Cutaneous Plasmacytosis with Perineural Involvement” [1], the authors incorrectly identified the ethnicity of the patient as Hispanic. The patient's correct ethnicity is Filipino.
